# No correlation between minimal electrical charge at the tip of the stimulating catheter and the efficacy of the peripheral nerve block catheter for brachial plexus block: a prospective blinded cohort study

**DOI:** 10.1186/1471-2253-14-26

**Published:** 2014-04-11

**Authors:** Karin PW Schoenmakers, Petra JC Heesterbeek, Nigel TM Jack, Rudolf Stienstra

**Affiliations:** 1 Department of Anesthesiology, Sint Maartenskliniek, Postbox 9011, 6500 GM Nijmegen, The Netherlands; 2 Research Department, Sint Maartenskliniek, Nijmegen, The Netherlands

**Keywords:** Peripheral nerve block, Nerve stimulation, Stimulating catheter

## Abstract

**Background:**

Stimulating catheters offer the possibility of delivering an electrical charge via the tip of the catheter. This may be advantageous as it allows verifying if the catheter tip is in close proximity to the target nerve, thereby increasing catheter performance. This prospective blinded cohort study was designed to investigate whether there is a correlation between the minimal electrical charge at the tip of the stimulating catheter, and the efficacy of the peripheral nerve block (PNB) catheter as determined by 24 h postoperative morphine consumption.

**Methods:**

Forty adult patients with ASA physical health classification I-III scheduled for upper extremity surgery under combined continuous interscalene block and general anesthesia were studied. Six patients were excluded from analysis.

After inserting a stimulating catheter as if it were a non-stimulating catheter for 2–5 cm through the needle, the minimal electrical charge necessary to obtain an appropriate motor response was determined. A loading dose of 20 mL ropivacaine 0.75% ropivacaine was then administered, and postoperative analgesia was provided by a continuous infusion of ropivacaine 0.2% 8 mL.h^-1^ via the brachial plexus catheter, and an intravenous morphine patient-controlled analgesia (PCA) device.

Main outcome measures include the minimal electrical charge (MEC) at the tip of the stimulating catheter necessary to elicit an appropriate motor response, and the efficacy of the PNB catheter as determined by 24 h postoperative PCA morphine consumption.

**Results:**

Mean (SD) [range] MEC at the tip of the stimulating catheter was 589 (1414) [30 – 5000] nC. Mean (SD) [range] 24 h morphine consumption was 8.9 (9.9) [0–29] mg. The correlation between the MEC and 24 h postoperative morphine consumption was Spearman’s Rho r_s_ = -0.26, 95% CI -0.56 to 0.09.

**Conclusion:**

We conclude that there is no proportional relation between MEC at the tip of the blindly inserted stimulating catheter and 24 h postoperative morphine consumption.

**Trial registration:**

Trialregister.nl identifier: NTR2328

## Background

Peripheral nerve block (PNB) is popular among anesthesiologists and patients for peri- and postoperative pain relief. PNB can be administered as a single shot or continuously using a catheter. For continuous PNB, non-stimulating and stimulating catheters are available. Non-stimulating catheters are inserted blindly through the needle after obtaining a correct needle position as determined by nerve stimulation (NS) and/or ultrasound. Because catheters are usually inserted some distance beyond the needle tip to avoid inadvertent dislocation, verifying a correct catheter position is not possible. Therefore, most anesthesiologists choose to administer a loading dose through the needle before placing the catheter. Whether the catheter tip is correctly placed does not become apparent until after the effect of the loading dose has worn off, usually late at night.

The use of ultrasound has become state of the art for PNB to ensure close proximity of the needle tip to the nerve before injecting the local anesthetic. Nevertheless, nerve stimulation is still widely used as the sole technique or to double-check needle position. Although there is no predefined relationship between the minimal electrical charge necessary to elicit an appropriate motor response, i.e. a contraction of a muscle innervated by the stimulated nerve (MEC), and the actual distance of the needle tip to the target nerve, it is generally assumed that the MEC has to be below 50 nanoCoulomb (nC) to ensure proximity close enough for effective nerve block [1,2] and above 20 nC to avoid inadvertent intraneural injection [3].

Stimulating catheters can be inserted while stimulating at the tip of the catheter. The expected added value of stimulation during insertion is that by maintaining an appropriate motor response, optimal positioning of the tip in close proximity of the nerve can be ensured. However, this is based on the assumption that an appropriate motor response with a sufficiently low electrical charge equals adequate positioning of the catheter tip. In other words: A low electrical charge necessary to evoke an appropriate motor response signals close proximity of the catheter tip to the nerve, whereas an increase in the MEC signals an increase in the distance between catheter tip and the nerve. Establishing a correct position of the catheter tip not only increases the likelihood of adequate postoperative analgesia, it also allows the administration of the loading dose fractionated through the catheter, thus reducing the risk of systemic toxicity. An obvious disadvantage is that stimulating catheters are more expensive and more needle manipulation may be necessary.

Recent literature has focused on the sensitivity of an appropriate motor response evoked by nerve stimulation in determining needle or catheter-nerve contact using ultrasonography as a reference [3-7]. However, these studies have focused on the false-negative response; i.e. no appropriate motor response in case of needle-nerve contact as visualized by ultrasound. When an appropriate motor response can be elicited with a low electrical charge, close proximity to the nerve is evident. However, when the necessary electrical charge is relatively high, or an appropriate motor response is absent, there are three possibilities: the tip of the catheter may either still be close enough to the nerve to provide adequate analgesia, or it may be at an intermediate distance with partial analgesic effect, or it may be too far off and inadequate for postoperative analgesia. One clinical way to evaluate if the tip of the catheter is adequately placed, is measuring postoperative morphine consumption: With a appropriately placed catheter tip, morphine consumption is expected to be low, whereas consumption is expected to increase if the catheter tip is farther off from the nerves.

One could hypothesize that the relation between MEC and morphine consumption is proportional, i.e. there is a linear correlation between the necessary electrical charge at the tip of the stimulating catheter and the adequacy of the catheter, justifying the extra manipulation to ensure close proximity to the nerve. The purpose of the present study is to investigate whether there is a correlation between the MEC at the tip of the blindly inserted stimulating catheter necessary to elicit an appropriate motor response, and the efficacy of the PNB catheter as determined by postoperative PCA morphine consumption. To investigate this hypothesis, we inserted a stimulating catheter as if it were a non-stimulating catheter and used the stimulation after placement as a measurement tool.

## Methods

### Ethics

Ethical approval for this study (Ethical Committee N° IRBN2009004) was provided by the Independent Review Board Nijmegen (Chairperson Dr. P. Koopmans) on 25 May 2009. This prospective blinded (for observer and patient) cohort study was registered at http://www.trialregister.nl (NTR2328) before onset of participant enrollment. Patients were informed about the study verbally and in writing and written informed consent was obtained from all patients. The study was conducted at the Sint Maartenskliniek Nijmegen, The Netherlands according to the Declaration of Helsinki and later revisions thereof and in accordance with the ICH guidelines for Good Clinical Practice.

### Patients

Patients scheduled for cuff-, stability repair or acromioplasty of the shoulder under continuous brachial plexus block were assessed for eligibility during the preoperative screening visit. Eligible participants were all adults aged 18 or over with ASA physical health classification I-III. None of the patients were known with a history of alcohol/drug dependence or abuse or with hepatic or renal insufficiency. Exclusion criteria included contra-indications for regional anesthesia (infection at the injection site, coagulopathy), known hypersensitivity to amide-type local anesthetics or opioids, known history of peripheral neuropathy, use of chronic analgesic therapy, and inability to understand numerical pain scores or to operate a Patient-Controlled Analgesia (PCA) device.

### Anesthetic procedure

Intravenous access and routine monitoring were established in all patients. Using ultrasound guidance (LOGIQ e 12 L-RS probe, GE Healthcare, Wauwatosa, USA), a short axis view, and in-plane approach, in combination with nerve stimulation, a 5 cm insulated Tuohy needle (Arrow, Teleflex Medical BV, Hilversum, The Netherlands) was inserted in the interscalene area by an anesthesiologist experienced in ultrasound-guided interscalene block. After obtaining a correct needle position as determined by ultrasound and a motor response of deltoid, triceps or biceps muscle with a stimulus below 50 nC (0.1 ms, < 0.5 mA), a stimulating catheter (Arrow StimuCath, Teleflex Medical BV, Hilversum, The Netherlands) was inserted 2–5 cm past the needle tip without stimulation; i.e. as if it were a non-stimulating catheter. We defined the MEC as the minimal electrical charge with which a motor response of a muscle innervated by the brachial plexus could be elicited. After determination of the MEC, brachial plexus block was established by injecting a total volume of 20 mL ropivacaine 0.75% in fractionated doses through the catheter. Time was designated t = 0 upon conclusion of the loading dose. Sensory block of the shoulder was assessed using loss of sensation to pin prick 30 min after injection if possible without compromising operating room (OR) logistics. Sensory block was scored as absent, partial or complete. Surgery was performed under general anesthesia with propofol, remifentanil and a laryngeal mask airway.

### Clinical assessments

After removal of the needle and fixation of the catheter and before administration of the loading dose, the MEC at the tip of the catheter necessary to evoke a motor response was determined and registered. If no response was present on the maximum current intensity of 1 mA at 0.1 ms, the pulse width was increased to 0.3 ms and then to 1.0 ms, the electrical charge thus varying from 0 to 1000 nC (nC = mA × ms × 1000); if no response was obtained at 1000 nC, the current scale was increased to 5 mA and a motor response was sought up to a maximum electrical charge of 5000 nC. The observer of motor response (KS) was blinded for the electrical charge.

One hour after administration of the brachial plexus loading dose, a continuous infusion of ropivacaine 0.2% 8 mL.h^-1^ was connected to the brachial plexus catheter and maintained until t = 24 h. Upon arrival in the recovery, the pain score (numerical rating scale: NRS 0–10) was noted and a PCA morphine device set up to deliver incremental doses of 1 mg of morphine with a lockout time of 5 minutes and no background infusion was connected to the intravenous cannula. Patients were instructed in the use of the PCA device preoperatively to maintain postoperative pain scores (NRS) at or below 3.

At t = 24 h, the continuous infusion through the catheter was stopped and the PCA device was disconnected by the investigator (KS). The total amount of administered morphine was registered. Patients were asked for their NRS at time of disconnection and their average and maximal NRS during the studied 24 h.

Primary outcome measures include the MEC necessary to evoke an appropriate motor response at the tip of the blindly inserted stimulating catheter and PCA morphine consumption during the first 24 h.

### Sample size and statistical analysis

In the absence of relevant data considering variation in electrical charge, we assumed ρ = 0.5 the smallest correlation to be relevant. The sample size needed for this correlation with α = 0.05 and a power of 0.9, was calculated to be 34 patients. To compensate for drop-out, we chose to include 40 patients in our study.

Data were analyzed using the GraphPad Prism 6 software (GraphPad Software Inc, San Diego, CA). Data are presented as mean (SD) [range] or proportions. Statistical analysis used the Spearman’s Rho for correlation coefficient calculation.

## Results

Forty patients were included. One patient showed symptoms of systemic toxicity (tinnitus, metallic taste) after 14 mL of ropivacaine 0.75% through the interscalene catheter. Injection was discontinued and the catheter was removed. The patient was treated with oxygen and prophylactic intravenous administration of lipid emulsion. No further treatment was necessary, symptoms resolving completely within a few minutes. Measured MEC at the tip of the catheter in this patient was 1425 nC.

The protocol was violated in another 5 patients. One patient mistakenly received a loading dose of 30 mL instead of 20 mL. In one patient the catheter was removed postoperatively because the patient was uncomfortable with it; later, this patient received an additional single shot interscalene block with 20 mL ropivacaine 0.2% because of pain; MEC in this patient was 72 nC. Three patients received an additional bolus of ropivacaine through the interscalene catheter immediately upon arrival at the recovery because of high pain scores. MEC values in these patients were 46, 68 and 120 nC respectively. These three patients had a complete sensory block of the shoulder prior to surgery. The six patients with protocol violations (Table 1) were excluded from subsequent analysis.

**Table 1:** Protocol violations

**Event**	MEC* (nC)
Toxic reaction after 14 mL loading dose ropivacaine 0.75%	1425
Loading dose of 30 instead of 20 mL ropivacaine 0.75%	46
Catheter discomfort and postoperative pain (catheter removed, single shot interscalene block with ropivacaine 0.2%)	72
Postop pain requiring extra ropivacaine (20 mL ropi 0.2%),	46
Postop pain requiring extra ropivacaine (10 mL ropi 0.75%)	68
Postop pain requiring extra ropivacaine (20 mL ropi 0.2%)	120

*MEC = Minimal electrical charge necessary to elicit an appropriate motor response.

Patient and surgical characteristics of the 34 patients in study are shown in Table 2.

**Table 2:** Patient and surgical characteristics

	**Total (n = 34)**
Sex; M/F	20/14
Age; years	49 (14)
BMI; kg.m^-2^	27 (4)
Duration of surgery; min	47 (16)
Type of surgery	Open rotator cuff repair (n = 12)
	Capsular shift (n = 11)
	Scopic acromioplasty (n = 4)
	Scopic rotator cuff repair (n = 2)
	Latissimus dorsi transfer (n = 2)
	Open acromioplasty (n = 1)
	Open Bankart repair (n = 1)
	Latarjet slap repair (n = 1)

Values are numbers or mean (SD).

All patients showed an appropriate motor response (deltoid, biceps or triceps muscle) during catheter stimulation, except for 2; one patient showed a phrenic nerve response and one patient had a response of the median nerve (finger flexion).

Sensory block of the shoulder at 30 min after injection could be assessed in 19 patients. In the remaining 15 patients surgery had already started before this time point. There were no postoperative complications related to the anesthetic procedure.

In three patients a different PCA device was mistakenly connected, with a continuous infusion of morphine 0.5 mg.h^-1^. Because these patients still required extra morphine boluses, they were not excluded from analysis. Total administered amount of morphine in these patients was 10, 23 and 27.7 mg; MEC values were 246, 38 and 60 nC respectively.

**Figure 1:**
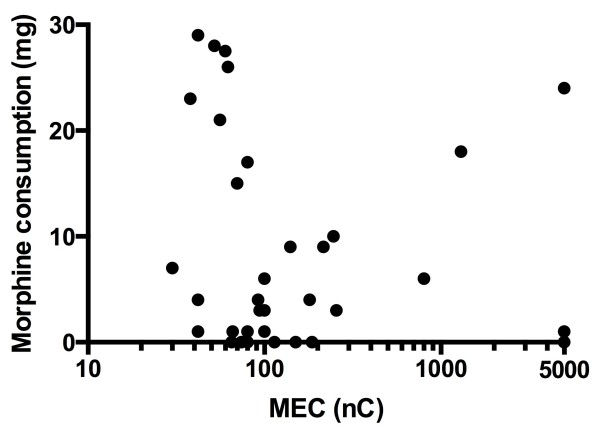
**Scatterplot of morphine consumption related to the electrical charge at the tip of the catheter in individual patients.** Note that the X-axis is not linear.

Mean morphine consumption was 8.9 (9.9) [0–29] mg (95% CI of the mean 5.4 to 12.3, n = 34). Data on individual parameters and clinical outcome measures are summarized in Tables 3 and 4 respectively. Spearman’s Rank Correlation Coefficient between the electrical charge (nC) at the tip of the catheter and morphine consumption was r_s_ = -0.26 (95% CI -0.56 to 0.09, n = 34). Figure 1 shows a scatterplot of morphine consumption as a function of the electrical charge at the tip of the catheter in individual patients.

**Table 3:** Individual outcome parameters

**M/F**	Response*	**Sensory block at 30 min**	NRS recovery**	**24 h morphine consumption; mg**	**MEC††; nC**
F	MD†	Complete	0	7	30
F	MD	Complete	0	23	38
M	MD	Unable¶	6	29	42
F	MB‡	Unable	2	4	42
M	MT§	Complete	0	1	42
M	MB	Complete	0	28	52
M	MT	Complete	4	21	56
F	MT	Complete	4	28	60
F	MD	Unable	0	26	62
M	MD	Partial	0	0	65
F	MT	Complete	0	1	66
F	MB	Unable	4	15	70
M	MB	Unable	0	0	74
M	MB	Unable	0	1	80
M	MT	Unable	0	17	80
M	MB	Unable	0	0	80
M	MT	Complete	0	4	92
F	MT	Complete	0	3	94
M	MD	Unable	0	1	100
M	MT	Unable	0	3	100
F	MT	Partial	0	6	100
M	MT	Complete	0	0	114
M	MB	Unable	0	9	140
M	MD	Complete	0	0	150
F	MB	Complete	0	4	180
M	MT	Complete	0	0	186
M	MT	Unable	0	9	216
F	Median nerve	Complete	5	10	246
F	MB	Unable	0	3	255
F	MD	Complete	0	6	800
F	MT	Unable	0	18	1300
M	MT	Partial	0	1	5000
M	MB	Unable	0	0	5000
M	Phrenic nerve	Partial	0	24	5000

*Response = motor response to nerve stimulation. †MD = Deltoid muscle; ‡MB = Biceps muscle; §MT = Triceps muscle. ¶Unable = Sensory block assessment at 30 min. not possible because patient already in surgery. **NRS recovery = numerical rating scale for pain upon arrival in the recovery room; ††MEC = Minimal electrical charge necessary to evoke an appropriate motor response.

**Table 4:** Clinical outcome measures

	**Total (n = 34)**
MEC (nC)	589 (1414) [30 – 5000]
NRS* recovery	0.7 (1.7) [0 – 6]
NRS t = 24 h	2.1 (2.1) [0 – 7]
NRS average during 24 h	2.5 (2.0) [0 – 7]
NRS max during 24 h	3.9 (2.8) [0 – 9]
Morphine consumption; mg	8.9 (9.9) [0 – 29]

Values are mean (SD) [range]. *NRS = numeric rating scale for pain.

## Discussion

The purpose of our study was to investigate the hypothesis that the relation between MEC and morphine consumption is proportional. We found no correlation between the minimal electrical charge at the tip of the stimulating catheter necessary to evoke an appropiate motor response and the efficacy of the PNB catheter for brachial plexus block.

The theoretical advantage of a stimulating catheter is that if stimulation of the catheter tip with a low charge elicits an appropriate motor response, correct catheter position at the time of stimulation may be assumed, although catheter tip migration at a later stage may of course still occur. One of the problems associated with continuous PNB is postoperative pain as a result of malfunctioning of the PNB catheter. The tip of the PNB catheter being too far away from the target nerves to establish effective pain relief may already occur during catheter insertion, or it may be caused by catheter tip migration during or after surgery. In our study the loading dose was administered through the catheter prior to surgery and the observation that four patients with a MEC less than 100 nC had pain scores at or above 4 upon arrival in the recovery room indicates that the interscalene block in these patients was insufficient despite a low MEC.

Stimulating catheters are more expensive than non-stimulating catheters, but the literature is mixed regarding the beneficial effects of the former. Some authors report advantages such as a shorter onset time, better postoperative analgesia or reduced postoperative local anesthetic and/or morphine consumption [8,9]. A semiquantitative systematic review found evidence for better postoperative analgesia with stimulating catheters [10]. Others fail to identify benefits of stimulating catheters and report no differences compared with traditional non-stimulating catheters [11,12]. Stevens et al. reported no differences in postoperative pain when comparing stimulating versus non-stimulating catheters for continuous interscalene block, but better functional outcome six weeks after surgery [13].

There is debate about the negative predictive value of nerve stimulation and the proximity of the needle tip tot the nerve. Perlas et al. noticed that occasionally a motor response to nerve stimulation up to 1.5 mA (150 nC) may be absent despite needle-nerve contact as observed by ultrasound [14]. In a study comparing the sensitivity of paresthesia and a motor response to nerve stimulation with an electrical charge of 50 nC or less, the sensitivity to nerve stimulation was 74.5% to detect needle-nerve contact as observed by ultrasound [6]. Using stimulating catheters and ultrasound as a reference, Altermatt et al. found that the sensitivity of an electrical charge of 50 nC to identify catheter-nerve contact was 64% [4]. Tsai et al. reported that with intraneural needle placement as determined by ultrasound, a motor response could be evoked with an average stimulus of 0.56 mA (56 nC), but in 12.5% of the cases the MEC ranged from 80–180 nC [3]. In a study quantifying the motor response with ultrasound-guided interscalene block, Sinha et al. found no differences in block characteristics between the stimulus eliciting a motor response being above or below 0.5 mA (50 nC) [15]. The absence of correlation between the MEC and postoperative morphine consumption found in this study indicates that the MEC has no predictive value about the postoperative efficacy of the PNB catheter.

The rationale for the extra cost of a stimulating catheter is in its positive predictive value, i.e. the association between an appropriate motor response following a MEC at or below a predefined level, and the incidence of a properly positioned PNB catheter as determined f.e. by postoperative PCA morphine consumption. The results of our study show that the positive predictive value of a lower MEC is not associated with a reduction in morphine consumption, indicating that the possibility of catheter tip stimulation does not result in a clinically relevant advantage. This indicates that the extra cost of a stimulating catheter is not balanced by a better efficacy of the catheter.

Our study has several limitations. Due to OR logistics, sensory block could not be assessed at 30 min in 15 patients because surgery had already started at that time. However, since the primary outcome parameter was the efficacy of the PNB catheter as determined by 24 h morphine consumption, verifying sensory block at 30 min was not strictly necessary. In addition, 12 of these 15 patients had an adequate sensory block upon arrival at the recovery, as judged by a NRS of 0.

PCA morphine consumption as a measure of PNB catheter efficacy may be criticised as it is an indirect tool at best; morphine consumption reflects the intensity of postoperative pain, but patients may use the PCA device for other discomforts as well. However, it is less time-consuming and less bothersome for patients than pin-prick assessments of sensory block at regular intervals, and in general the relation between morphine consumption and postoperative pain will be proportional.

It may be argued that we did not investigate PNB catheter efficacy per se because the effect of the loading dose alone will last 8–12 h, but may last as long as 20 h and we measured morphine consumption only up to 24 h. However, since the loading dose was administered through the PNB catheter after positioning the catheter and determining the MEC, we believe that our findings adequately reflect the relation between MEC and catheter efficacy.

## Conclusion

In conclusion, our results show that in interscalene brachial plexus block the MEC at the catheter tip necessary to evoke an appropriate motor response has no correlation with catheter efficacy as determined by postoperative 24 h morphine consumption.

## Competing interests

All authors declare to have no financial or non-financial competing interests.

## Authors’ contributions

KS conceived of the study, participated in its design, carried out the study and drafted the manuscript. PH performed the randomisation for the study and helped with the statistical analysis. NJ participated in the design of the study and corrected the language. RS conceived of the study, participated in its design and coordination and helped to draft the manuscript. All authors read and approved the final manuscript.

## Pre-publication history

The pre-publication history for this paper can be accessed here:

http://www.biomedcentral.com/1471-2253/14/26/prepub
